# Supplementary material for: Aire-dependent genes undergo Clp1-mediated 3’UTR shortening associated with higher transcript stability in the thymus
Source: eLife. 2020 Apr 29;9:e52985. doi: 10.7554/eLife.52985 (PMC7205469; doi:10.7554/eLife.52985)
Supplement: Figure 1—source data 1. — A proximal pA was validated when its genomic location from the PolyA_DB 2 database differs from 20 bp at least to the genomic location of the UCSC annotated 3’UTR distal boundary or distal pA. In case of multiple proximal pAs, the most proximal one was considered. The two annotation files (GFF2_features_hg19_UTR_d.gtf and GFF2_features_mm9_UTR_d.gtf) are GTF files to be used with intersectBed and coverageBed for RNA-seq-based d3’UTR ratio calculation. The two annotation files (features_hg19_UTR_d.csv and features_mm9_UTR_d.csv) are to be used with our R-implementation of PLATA (Giraud et al., 2012) for individual probe-level microarray analyses and microarray-based d3’UTR ratio calculation. [file elife-52985-fig1-data1.zip › Figure_1_source_data_1_REVISION/Figure 1ΓÇôsource data 1.docx]

**Figure 1–source data 1. d3’UTR annotation files in mice and humans for RNA-seq and microarray analyses**

A proximal pA was validated when its genomic location from the PolyA_DB 2 database differs from 20bp at least to the genomic location of the UCSC annotated 3’UTR distal boundary or distal pA. In case of multiple proximal pAs, the most proximal one was considered.

GFF2_features_hg19_UTR_d.gtf

GFF2_features_mm9_UTR_d.gtf

These two annotation files are GTF files to be used with intersectBed and coverageBed for RNA-seq-based d3’UTR ratio calculation.

features_hg19_UTR_d.csv

features_mm9_UTR_d.csv

These two annotation files are to be used with our R-implementation of PLATA (Giraud et al. 2012) for individual probe-level microarray analyses and microarray-based d3’UTR ratio calculation.
