# Supplementary material for: Aire-dependent genes undergo Clp1-mediated 3’UTR shortening associated with higher transcript stability in the thymus
Source: eLife. 2020 Apr 29;9:e52985. doi: 10.7554/eLife.52985 (PMC7205469; doi:10.7554/eLife.52985)
Supplement: Figure 1—source data 2. — The two annotation files (UCSCmm9.gtf and UCSChg19.gtf) are GTF files to be used with intersectBed and coverageBed for RNA-seq differential gene expression analyses. [file elife-52985-fig1-data2.zip › Figure_1_source_data_2_REVISION/Figure 1ΓÇôsource data 2.docx]

**Figure 1–source data 2. Annotation files in mice and humans for RNA-seq differential gene expression.**

UCSCmm9.gtf

UCSChg19.gtf

These two annotation files are GTF files to be used with intersectBed and coverageBed for RNA-seq differential gene expression analyses
