# Supplementary material for: Aire-dependent genes undergo Clp1-mediated 3’UTR shortening associated with higher transcript stability in the thymus
Source: eLife. 2020 Apr 29;9:e52985. doi: 10.7554/eLife.52985 (PMC7205469; doi:10.7554/eLife.52985)
Supplement: Figure 1—source data 3. — RNAseq_d3UTR_ratio_density.R: R-script used for d3’UTR ratio calculation and d3’UTR ratio density plot from RNA-seq data. The script needs the WT_AireKO_counts_UCSCmm9_DESEQ.csv file (DESeq differential expression file of WT vs Aire-KO mTEChi), the WT_AireKO_counts_UTRd.csv file (count of reads mapping the annotated features including d3’UTRs: GFF2_features_mm9_UTR_d.gtf provided in Figure 1—source data 1) and the number of reads of the WT and Aire-KO samples that map to the mm9 genome (114828554 for WT mTEChi and 179098324 for Aire-KO mTEChi). [file elife-52985-fig1-data3.zip › Figure_1_source_data_3_REVISION/Figure 1ΓÇôsource data 3.docx]

**Figure 1–source data 3. d3’UTR ratio calculation in WT and *Aire*-KO mTEChi.**

RNAseq_d3UTR_ratio_density.R:

R-script used for d3’UTR ratio calculation and d3’UTR ratio density plot from RNA-seq data. The script needs the WT_AireKO_counts_UCSCmm9_DESEQ.csv file (DESeq differential expression file of WT vs *Aire*-KO mTEChi), the WT_AireKO_counts_UTRd.csv file (count of reads mapping the annotated features including d3’UTRs: GFF2_features_mm9_UTR_d.gtf provided in **Figure 1-source data 1**) and the number of reads of the WT and *Aire*-KO samples that map to the mm9 genome.

Dependent files:

WT_AireKO_counts_UCSCmm9_DESEQ.csv

WT_AireKO_counts_UTRd.csv

Number of reads mapping WT mTEChi: 114828554

Number of reads mapping Aire-KO mTEChi: 179098324
