# Supplementary material for: Aire-dependent genes undergo Clp1-mediated 3’UTR shortening associated with higher transcript stability in the thymus
Source: eLife. 2020 Apr 29;9:e52985. doi: 10.7554/eLife.52985 (PMC7205469; doi:10.7554/eLife.52985)
Supplement: Figure 2—source data 1. — The four files (3UTRd+_const.xlsx, 3UTRd+_alt.xlsx, 3UTRd-_const.xlsx and 3UTRd-_alt.xlsx) were generated parsing the PolyA_DB 2 database for alternative (proximal) pAs and constitutive (distal) pAs. Alternative pAs with their genomic location are listed in two files according to the orientation of the genes (3UTRd+_alt.xlsx and 3UTRd-_alt.xlsx). Constitutive pAs with their genomic location are also listed in two different files (3UTRd+_const.xlsx and 3UTRd-_const.xlsx). These files are used to generate bed files of proximal and distal pA location at Aire-sensitive and neutral-genes for CLIP-seq analysis using the sitepro program (CEAS distribution). [file elife-52985-fig2-data1.zip › Figure_2_source_data_1_REVISION/Figure 2ΓÇôsource data 1.docx]

**Figure 2–source data 1. Genomic location of pAs on hg19 extracted from the PolyA_DB 2 database for CLIP-seq analysis.**

3UTRd+_const.xlsx

3UTRd+_alt.xlsx

3UTRd-_const.xlsx

3UTRd-_alt.xlsx

These four files were generated parsing the PolyA_DB 2 database for alternative (proximal) pAs and constitutive (distal) pAs. Alternative pAs with their genomic location are listed in two files according to the orientation of the genes (3UTRd+_alt.xlsx and 3UTRd-_alt.xlsx). Constitutive pAs with their genomic location are also listed in two different files (3UTRd+_const.xlsx and 3UTRd-_const.xlsx).

These files are used to generate bed files of proximal and distal pA location at Aire-sensitive and neutral-genes for CLIP-seq analysis using the sitepro program (CEAS distribution).
