# Supplementary material for: Aire-dependent genes undergo Clp1-mediated 3’UTR shortening associated with higher transcript stability in the thymus
Source: eLife. 2020 Apr 29;9:e52985. doi: 10.7554/eLife.52985 (PMC7205469; doi:10.7554/eLife.52985)
Supplement: Figure 3—source data 1. — We use the aroma.affymetrix R-package and the commands listed in the aroma.affymetrix.commands.docx file for ‘probeset expression extraction’ and ‘individual probe expression extraction’. A mandatory folder organization associated with this package is needed for the analysis. The sample CEL files must be added in a dedicated folder. (HuGene-1_0 st-v1,r3.cdf) is the Chip Description File. (probeset.csv) is the list of probeset IDs corresponding to the genes on the array. (HuGeneST1features.csv) is the list of probeset IDs and their individual probe IDs. [file elife-52985-fig3-data1.zip › Figure_3_source_data_1_REVISION/aroma.affymetrix.commands.docx]

**For probeset expression extraction:**

cdf <- AffymetrixCdfFile$byChipType("HuGene-1_0-st-v1,r3")

cs <- AffymetrixCelSet$byName("monset", cdf=cdf)

bc <- RmaBackgroundCorrection(cs)

csBC <- process(bc,verbose=verbose)

qn <- QuantileNormalization(csBC, typesToUpdate="pm")

csN <- process(qn, verbose=verbose)

plm <- RmaPlm(csN)

fit(plm)

ces <- getChipEffectSet(plm)

theta <- extractMatrix(ces)

rownames(theta) <- getUnitNames(cdf)

write.csv(theta,"final.csv") *# associate “probeset.csv” with “final.csv”*

**For individual probe expression extraction:**

cdf <- AffymetrixCdfFile$byChipType("HuGene-1_0-st-v1,r3")

cs <- AffymetrixCelSet$byName("monset", cdf=cdf)

bc <- RmaBackgroundCorrection(cs)

csBC <- process(bc,verbose=verbose)

qn <- QuantileNormalization(csBC, typesToUpdate="pm")

csN <- process(qn, verbose=verbose)

temp<-csN$intensities

r<-row(temp)

temp2<-cbind(r[,1],temp)

dat<-read.csv("HuGeneST1features.csv",header=FALSE)

sup<-temp2[match(dat[,5],temp2[,1]),2:5]

*# adjust the range 2:5 according to the number of sample CEL files*

datTOUT<-cbind(dat[,3:5],sup)

colnames(datTOUT)<-c("probeset","indice","indices",…)

*# ”...” to complete with the sample names*

write.csv(datTOUT,file="HuGeneST1features_SAMPLE_TOUT.csv",row.names=FALSE)
