# Supplementary material for: Aire-dependent genes undergo Clp1-mediated 3’UTR shortening associated with higher transcript stability in the thymus
Source: eLife. 2020 Apr 29;9:e52985. doi: 10.7554/eLife.52985 (PMC7205469; doi:10.7554/eLife.52985)
Supplement: Figure 3—source data 1. — We use the aroma.affymetrix R-package and the commands listed in the aroma.affymetrix.commands.docx file for ‘probeset expression extraction’ and ‘individual probe expression extraction’. A mandatory folder organization associated with this package is needed for the analysis. The sample CEL files must be added in a dedicated folder. (HuGene-1_0 st-v1,r3.cdf) is the Chip Description File. (probeset.csv) is the list of probeset IDs corresponding to the genes on the array. (HuGeneST1features.csv) is the list of probeset IDs and their individual probe IDs. [file elife-52985-fig3-data1.zip › Figure_3_source_data_1_REVISION/Figure 3ΓÇôsource data 1.docx]

**Figure 3–source data 1. Human Gene ST1.0 microarray probeset and individual probe expression extraction.**

HuGene-1_0-st-v1,r3.cdf the Chip Description File

probeset.csv the list of probeset IDs corresponding to the genes on the array

HuGeneST1features.csv the list of probeset IDs and their individual probe IDs

We use the aroma.affymetrix R-package and the commands listed in aroma.affymetrix.commands.docx for “probeset expression extraction” and “individual probe expression extraction”. A mandatory folder organization associated with this package is needed for the analysis. The sample CEL files must be added in a dedicated folder.
