# Supplementary material for: Aire-dependent genes undergo Clp1-mediated 3’UTR shortening associated with higher transcript stability in the thymus
Source: eLife. 2020 Apr 29;9:e52985. doi: 10.7554/eLife.52985 (PMC7205469; doi:10.7554/eLife.52985)
Supplement: Figure 3—source data 2. — Microarray_d3UTR_hg19.R: R-script to perform individual probe d3’UTR mapping and d3’UTR ratio calculation. Dependent files: - annotation file including 3d’UTR features for microarray analysis (Figure 1—source data 1): features_hg19_UTR_d.csv - Individual probe location on hg19: HuGene-1_0 st-v1.hg19.probe.csv - Individual probe expression obtained from the comparison: CTR versus CLP1 sh2 KD samples (Figure 3—source data 1): ST1features_CTR_v_CLP1_SH2.csv Result file: global_HuGene_CTR_v_CLP1_SH2.csv In the result file, the feat_WTnorm and feat_KOnorm columns correspond, for the 3UTRd features, to the d3’UTR ratios in WT and KO samples, respectively. [file elife-52985-fig3-data2.zip › Figure_3_source_data_2_REVISION/Figure 3ΓÇôsource data 2.docx]

**Figure 3–source data 2. Microarray individual probe d3’UTR mapping and d3’UTR ratio calculation.**

- R-script to perform individual probe d3’UTR mapping and d3’UTR ratio calculation:

Microarray_d3UTR_hg19.R

Dependent files:

- annotation file including 3d’UTR features for microarray analysis (**Figure 1-source data 1**):

features_hg19_UTR_d.csv

- Individual probe location on hg19:

HuGene-1_0-st-v1.hg19.probe.csv

- Individual probe expression obtained from the comparison: CTR versus CLP1 sh2 KD samples (**Figure 3-source data 1**):

ST1features_CTR_v_CLP1_SH2.csv

Result file:

global_HuGene_CTR_v_CLP1_SH2.csv

In the result file, the feat_WTnorm and feat_KOnorm columns correspond, for the 3UTRd features, to the d3’UTR ratios in WT and KO samples, respectively.
