# Supplementary material for: Aire-dependent genes undergo Clp1-mediated 3’UTR shortening associated with higher transcript stability in the thymus
Source: eLife. 2020 Apr 29;9:e52985. doi: 10.7554/eLife.52985 (PMC7205469; doi:10.7554/eLife.52985)
Supplement: Figure 3—source data 3. — Microarray_d3UTR_significance.R: R-script used to perform the analysis. (global_HuGene_CTR_v_CLP1_SH2_3dUTR.csv) corresponds to global_HuGene_CTR_v_CLP1_SH2.csv that was obtained from Figure 3—source data 2 and restricted to the d3’UTR features. [file elife-52985-fig3-data3.zip › Figure_3_source_data_3_REVISION/Figure 3ΓÇôsource data 3.docx]

**Figure 3–source data 3. d3’UTR ratio imbalance obtained from microarray data.**

Microarray_d3UTR_significance.R: R-script used to perform the analysis

global_HuGene_CTR_v_CLP1_SH2_3dUTR.csv: This file corresponds to: global_HuGene_CTR_v_CLP1_SH2.csv that was obtained from **Figure 3-source data 2** and restricted to the d3’UTR features.
