# Supplementary material for: Aire-dependent genes undergo Clp1-mediated 3’UTR shortening associated with higher transcript stability in the thymus
Source: eLife. 2020 Apr 29;9:e52985. doi: 10.7554/eLife.52985 (PMC7205469; doi:10.7554/eLife.52985)
Supplement: Figure 5—source data 1. — In both files (miRNA_target_Refseq.gmt and miRNA_target_Refseq_d3UTR_filtered.csv), the first two columns show miRNA families and individual miRNA(s). (miRNA_target_Refseq.gmt) was used in GSEA to determine the miRNA-specific target gene sets showing significant enrichment in mTEChi or mTEClo. (miRNA_target_Refseq_d3UTR_filtered.csv) was used to determine the proportion of Aire-sensitive and neutral genes potentially targeted in their d3’UTRs by miRNAs. The file (Leading_edge_mTEChi_vs_lo_346_miRNAs.xlsx) summarizes the leading edge analysis of 346 miRNAs or miRNA families and lists the core targets that account for enrichment signals of gene sets significantly deviated (FDR < 0.05) in mTEChi versus mTEClo. Here all significant gene sets are reduced in mTEChi (enriched in mTEClo). Table values are rank metric scores. The last line of the table shows the number of miRNAs or miRNA families potentially recognizing specific leading edge targets. [file elife-52985-fig5-data1.zip › Figure_5_source_data_1_REVISION/Figure 5ΓÇôsource data 1.docx]

**Figure 5–source data 1. Potential miRNA-specific target genes with conserved miRNA sites in their 3’UTRs or d3’UTRs, from the TargetScan 6.2 database.**

miRNA_target_Refseq.gmt

miRNA_target_Refseq_d3UTR_filtered.csv

In both files, the first two columns show miRNA families and individual miRNA(s).

miRNA_target_Refseq.gmt was used in GSEA to determine the miRNA-specific target gene sets showing significant enrichment in mTEChi or mTEClo.

miRNA_target_Refseq_d3UTR_filtered.csv was used to determine the proportion of Aire-sensitive and neutral genes potentially targeted in their d3’UTRs by miRNAs.

Leading_edge_mTEChi_vs_lo_346_miRNAs.xlsx

This file summarizes the leading edge analysis of 346 miRNAs or miRNA families and lists the core targets that account for enrichment signals of gene sets significantly deviated (FDR<0.05) in mTEChi versus mTEClo. Here all significant gene sets are reduced in mTEChi (enriched in mTEClo). Table values are rank metric scores. The last line of the table shows the number of miRNAs or miRNA families potentially recognizing specific leading edge targets.
