# Supplementary material for: Aire-dependent genes undergo Clp1-mediated 3’UTR shortening associated with higher transcript stability in the thymus
Source: eLife. 2020 Apr 29;9:e52985. doi: 10.7554/eLife.52985 (PMC7205469; doi:10.7554/eLife.52985)
Supplement: Supplementary file 1. [file elife-52985-supp1.docx]

| **Gene** | **Refseq** | **Clone ID** | **Ranked sh** | **KD %remaining** |
| --- | --- | --- | --- | --- |
| *CLP1* | NM_006831 | TRCN0000015039 | **sh1** | **23** |
|  |  | TRCN0000329955 | **sh2** | **25** |
|  |  | TRCN0000329954 | **sh3** | **28** |
|  |  | TRCN0000353578 | sh4 | 29 |
|  |  | TRCN0000353577 | sh5 | 32 |
|  |  | TRCN0000329956 | sh6 | 68 |
| *Clp1* | NM_133840 | TRCN0000084982 | **sh1** | **31** |
|  |  | TRCN0000304342 | **sh2** | **39** |
|  |  | TRCN0000084980 | **sh3** | **51** |
|  |  | TRCN0000310922 | sh4 | 134 |
| *HNRNPL* | NM_001533 | TRCN0000221617 | **sh1** | **21** |
|  |  | TRCN0000330564 | **sh2** | **26** |
|  |  | TRCN0000330620 | **sh3** | **28** |
|  |  | TRCN0000221618 | sh4 | 42 |
|  |  | TRCN0000330565 | sh5 | 43 |
| *DDX5* | NM_004396 | TRCN0000272491 | **sh1** | **22** |
|  |  | TRCN0000272488 | **sh2** | **26** |
|  |  | TRCN0000272434 | **sh3** | **43** |
|  |  | TRCN0000272436 | sh4 | 58 |
|  |  | TRCN0000001128 | sh5 | 65 |
|  |  | TRCN0000001127 | sh6 | 99 |
| *DDX17* | NM_006386 | TRCN0000074390 | **sh1** | **20** |
|  |  | TRCN0000286979 | **sh2** | **26** |
|  |  | TRCN0000294332 | **sh3** | **30** |
|  |  | TRCN0000287045 | sh4 | 44 |
| *PARP1* | NM_001618 | TRCN0000007929 | **sh1** | **16** |
|  |  | TRCN0000338467 | **sh2** | **22** |
|  |  | TRCN0000007930 | **sh3** | **23** |
|  |  | TRCN0000356475 | sh4 | 25 |
|  |  | TRCN0000007928 | sh5 | 38 |
|  |  | TRCN0000007931 | sh6 | 44 |
| *SUPT16H* | NM_007192 | TRCN0000293349 | **sh1** | **3** |
|  |  | TRCN0000293281 | **sh2** | **6** |
|  |  | TRCN0000293350 | **sh3** | **11** |
|  |  | TRCN0000293348 | sh4 | 12 |
|  |  | TRCN0000001260 | sh5 | 28 |
|  |  | TRCN0000293313 | sh6 | 42 |
| *PRKDC* | NM_006904 | TRCN0000195491 | **sh1** | **13** |
|  |  | TRCN0000194719 | **sh2** | **14** |
|  |  | TRCN0000197152 | **sh3** | **15** |
|  |  | TRCN0000194985 | sh4 | 27 |
|  |  | TRCN0000006256 | sh5 | 31 |
|  |  | TRCN0000006258 | sh6 | 54 |
| *PABPC1* | NM_002568 | TRCN0000293649 | **sh1** | **8** |
|  |  | TRCN0000074639 | **sh2** | **13** |
|  |  | TRCN0000293599 | **sh3** | **21** |
|  |  | TRCN0000074642­ | sh4 | 87 |
| *CPSF6* | NM_007007 | TRCN0000237833 |  | **36** |
| lacZ | lacZ.1 | TRCN0000072240 |  |  |

**Supplementary File 1. List of shRNAs**
