# Supplementary material for: Aire-dependent genes undergo Clp1-mediated 3’UTR shortening associated with higher transcript stability in the thymus
Source: eLife. 2020 Apr 29;9:e52985. doi: 10.7554/eLife.52985 (PMC7205469; doi:10.7554/eLife.52985)
Supplement: Supplementary file 2. [file elife-52985-supp2.docx]

| **Specie** | **Target** | **Forward** | **Reverse** |
| --- | --- | --- | --- |
| Human | *CLP1* | gagtttacctgttgggctcct | tggttggcttcttgtcatca |
| Mouse | *Clp1* | gcaccaacatcaagctttacaat | gacccagccacaggtgtt |
| Human | *HNRNPL* | ggaggtgaccgaggagaact | cgctcacttttgcctgaga |
| Human | *DDX5* | gccatgtcgggttattcg | ggtttccaaacttctttccaga |
| Human | *DDX17* | ccatacttggaaaggggagat | gtacttgctgggcaagctct |
| Human | *PARP1* | tggaggacgacaaggaaaac | tgttgctaccgatcaccgta |
| Human | *SUPT16H* | tttagggacttgggatttaacg | tccaatgtcaccacaaaagg |
| Human | *PRKDC* | cattggcgagagaactgtagg | gccactgctttcaaaagtga |
| Human | *PABPC1* | tgtactacaagcccaccaagc | tcatggtccctgatcaatttt |
| Human | *CPSF6* | ggtggggacagatttcctg | gtggacgtggtggagtctg |
| Human | *GAPDH* | agccacatcgctcagacac | gcccaatacgaccaaatcc |
| Mouse | *Gapdh* | ggcaaattcaacggcacagt | agatggtgatgggcttccc |
|  | TetOn3G | ctatgcccccacttctgaaa | gtcagcaggcagcatatcaa |
|  | ZsGreen | gtcagcttgtgctggatgaa | ccccgtgatgaagaagatga |

**Supplementary File 2. List of primers**
