# Supplementary material for: Aire-dependent genes undergo Clp1-mediated 3’UTR shortening associated with higher transcript stability in the thymus
Source: eLife. 2020 Apr 29;9:e52985. doi: 10.7554/eLife.52985 (PMC7205469; doi:10.7554/eLife.52985)
Supplement: Transparent reporting form [file elife-52985-transrepform.pdf]

eLIFE
1st Floor
24 Hills Road
Cambridge CB2 1JP, UK
P 01223 855340
W elifesciences.org
T @elife

eLife's transparent reporting form

We encourage authors to provide detailed information *within their submission* to facilitate the interpretation and replication of experiments. Authors can upload supporting documentation to indicate the use of appropriate reporting guidelines for health-related research (see EQUATOR Network), life science research (see the BioSharing Information Resource), or the ARRIVE guidelines for reporting work involving animal research. Where applicable, authors should refer to any relevant reporting standards documents in this form.

If you have any questions, please consult our Journal Policies and/or contact us: editorial@elifesciences.org.

Sample-size estimation

- You should state whether an appropriate sample size was computed when the study was being designed
- You should state the statistical method of sample size computation and any required assumptions
- If no explicit power analysis was used, you should describe how you decided what sample (replicate) size (number) to use

Please outline where this information can be found within the submission (e.g., sections or figure legends), or explain why this information doesn't apply to your submission:

For lentigenic mouse generation, previous experiments that we performed at Harvard Medical School in the lab of Christophe Benoist and Diane Mathis (2010-2011) (Giraud et al. 2014), showed that approximately one third to one half of oocytes transduced with an ultra high-titer PLKO shRNA lentivirus led to viable pups with the PLKO construct properly integrated into the genome of mTEChi. These previous experiments also showed that the integration of the lentiviral construct does not necessarily result in the expression of its bearing-shRNA due to the integration site and variegation. We estimated that about one fifth of the generated pups, which have shRNA integrated into the mTEChi genome, show shRNA expression in these cells.

Based on these data, we reimplanted 33 transduced oocytes into five pseudopregnant females and obtained 19 viable pups. Consistent with our estimations, two pups were expressing Clp1-specific shRNAs in mTEChi and one was showing a >50% reduction (60%) of the Clp1 mRNA levels (**Mat and Meth – Lentigenic mouse generation** and **Main text – Clp1 promotes 3'UTR shortening and higher levels of Aire-upregulated transcripts in mTEChi**).

Replicates

- You should report how often each experiment was performed
- You should include a definition of biological versus technical replication
- The data obtained should be provided and sufficient information should be provided to indicate the number of independent biological and/or technical replicates
- If you encountered any outliers, you should describe how these were handled
- Criteria for exclusion/inclusion of data should be clearly stated

eLife Sciences Publications, Ltd is a limited liability non-profit non-stock corporation incorporated in the State of Delaware, USA, with company number 5030732, and is registered in the UK with company number FC030576 and branch number BR015634 at the address 1st Floor, 24 Hills Road, Cambridge CB2 1JP | August 2014

- High-throughput sequence data should be uploaded before submission, with a private link for reviewers provided (these are available from both GEO and ArrayExpress)

Please outline where this information can be found within the submission (e.g., sections or figure legends), or explain why this information doesn't apply to your submission:

- WT and Aire-KO mTEChi transcript profiling were carried out in cells sorted from pools of thymi isolated from 4 WT and 4 Aire-KO B6 mice (**legend of Figure 1 and Figure 1–figure supplement 1**). Two independent experiments generating two independent WT and Aire-KO RNAseq datasets were performed. The two datasets were analyzed separately.

- HEK293 cells that we used to identified HEK293-specific Aire-sensitive genes and their 3'UTR lengthening after Aire-transfection were those that Martin et al. 2012 used to generate CSTF2 PAR_CLIP sequencing data. They kindly provided us with these cells and we could re-analyze their PAR_CLIP data in regard to the genes that we found to be Aire-sensitive in these cells.

- We also used these HEK293 cells for shRNA knockdown experiments. shRNA-triggered knockdown efficiency was tested after lenti-infection of two independent HEK293 cell cultures (biological duplicates) for human shRNAs or 1C6 cells for murine shRNAs. Quantitative RT-PCR of each shRNA-target gene was carried out in triplicate (technical triplicates) in each sample (**Mat and Meth – shRNA-mediated knockdown**). Hit shRNAs triggering more than 50% reduction of their target gene mRNA levels were selected.

- In HEK293 cells, the effect resulting from the knockdown of a target gene was confirmed when at least two independent experiments by two different hit shRNAs of the same target gene yielded similar results, therefore ruling out non-specific off-target confounding effect (**Main text – CLP1 promotes 3'UTR shortening and higher expression at Aire-sensitive genes in HEK293 cells**).

- To increase the chances to get an efficient Clp1-knockdown in lentigenic mTEChi, three hit Clp1-specific shRNAs validated in 1C6 cells were cloned together in a lentiviral vector that we used to microinfect fertilized oocytes (**Mat and Meth – Lentigenic mouse generation**).

- Microarray and RNAseq profiling of Ctr and knockdown HEK293 cells or mTEChi were carried out in single samples and analyzed at gene set levels.

- Gene expression profiling in Ctr or actinomycin D-treated mTEChi was carried out in a time series format by RNAseq of mTEChi sorted from pooled thymi of B6 mice (**Mat and Meth – Actinomycin D treatment**).

Statistical reporting

- Statistical analysis methods should be described and justified
- Raw data should be presented in figures whenever informative to do so (typically when N per group is less than 10)
- For each experiment, you should identify the statistical tests used, exact values of N, definitions of center, methods of multiple test correction, and dispersion and precision measures (e.g., mean, median, SD, SEM, confidence intervals; and, for the major substantive results, a measure of effect size (e.g., Pearson's r, Cohen's d)
- Report exact p-values wherever possible alongside the summary statistics and 95% confidence intervals. These should be reported for all key questions and not only when the p-value is less than 0.05.

Please outline where this information can be found within the submission (e.g., sections or figure legends), or explain why this information doesn't apply to your submission:

- Genes sensitive to the upregulation by Aire were identified by differential expression in WT vs Aire-KO mTEChi and in Aire-transfected vs Ctr-transfected HEK293 cells using expression foldchange thresholds above which the genes exhibit significant foldchange Z scores ($P<0.01$) (**legend of Figure 1 and Figure 2–figure supplement 1**)

- The error bars for the d3'UTR ratios or expression values correspond to 95% confidence interval of the medians. (**legend of most Figures and figure supplements**)

- Determination of the statistical significance for quantitative differences (ex: d3'UTR ratio, expression value, expression foldchange) between two experimental groups was performed using the non-parametric Wilcoxon test. When appropriate, and to avoid inflation of significance level due to a large number of items in one group of the comparison, we selected a number of Aire-neutral genes equal to the number of Aire-sensitive genes (**legend of most Figures and figure supplements, and Mat and Meth – Statistical analysis**)

- For qualitative differences such as the d3'UTR ratio imbalance in microarray data, we performed Chi-squared tests (**legend of Figure 3**)

(For large datasets, or papers with a very large number of statistical tests, you may upload a single table file with tests, Ns, etc., with reference to sections in the manuscript.)

Group allocation

- Indicate how samples were allocated into experimental groups (in the case of clinical studies, please specify allocation to treatment method); if randomization was used, please also state if restricted randomization was applied
- Indicate if masking was used during group allocation, data collection and/or data analysis

Please outline where this information can be found within the submission (e.g., sections or figure legends), or explain why this information doesn't apply to your submission:

When possible, pools of 4 thymi isolated from B6 mice were used to obtain sufficient number of mTEChi to perform Illumina sequencing without extra rounds of cDNA preamplification. 30 000 mTEChi can be isolated from one thymus. Hence pools of 4 thymi enabled to sort more than 100 000 mTEChi and to use the Illumina TruSeq sequencing chemistry. Using pooled mTEChi also reduces potential inter-individual heterogeneity.

Additional data files ("source data")

- We encourage you to upload relevant additional data files, such as numerical data that are represented as a graph in a figure, or as a summary table
- Where provided, these should be in the most useful format, and they can be uploaded as "Source data" files linked to a main figure or table
- Include model definition files including the full list of parameters used
- Include code used for data analysis (e.g., R, MatLab)
- Avoid stating that data files are "available upon request"

Please indicate the figures or tables for which source data files have been provided:

- Source data corresponding to the selection of Aire-sensitive genes with proximal pAs in WT vs Aire-KO mTEChi is provided in (**Figure 1–source data 2**)

- Source data corresponding to Aire-sensitive genes with proximal pAs in Ctr versus Clp1 KD mTEChi is provided in (**Figure 4–source data 1**)

- The code that we used for microarray analyses at the individual probe level and that we wrote as an R-implementation of the PLATA algorithm (Sandberg et al. 2008) has been published and is available in (Giraud et al. 2012)

- The d3'UTR annotation files that we generated in mice and humans in parsing the PolyA_DB 2 database for individual probe level microarray and RNAseq analyses are provided in (**Figure 1–source data 1**)
